# OncoPSM: an interactive tool for cost-effectiveness analysis using partitioned survival models in oncology trial

**DOI:** 10.3389/fphar.2025.1554405

**Published:** 2025-08-26

**Authors:** Xulong Qiu, Jidi Chen, Muting Wang, Kaixin Zheng, Ruixiong Li

**Affiliations:** ^1^ Department of Cardiothoracic Surgery, Shantou Central Hospital, Shantou, China; ^2^ Medical College, Shantou University, Shantou, China

**Keywords:** cost-effectiveness analysis, incremental cost-effectiveness ratio, partitioned survival model, oncology, lung cancer

## Abstract

**Introduction:**

Cost-effectiveness analysis (CEA) serves as a critical tool to evaluate the economic sustainability of new treatments. However, many CEA tools are not specifically tailored to address the intricate cost composition resulting from the complex treatment regimens in oncology trials.

**Methods:**

We extracted data from Kaplan-Meier (KM) curves, reconstructed individual patient data (IPD) using an iterative KM algorithm, and fitted parametric survival functions to the IPD data. Based on these functions, we constructed Partitioned Survival Model (PSM), calculated the probability of each survival state per cycle, and combined these with utility values to compute the effect per cycle and the incremental effect for the experimental group. We employed a treatment-cycle-specific cost analysis, simulating cost uncertainty through gamma distribution. Using the PSM, we calculated the state-weighted cost, applied a discount rate, determined the incremental cost for the experimental group, and calculated the Incremental Cost-Effectiveness Ratio (ICER).

**Results:**

The OncoPSM application is available at http://sw2-primary1.xiyoucloud.pro:13471/oncoPSM/. Validation with real-world data from the CHOICE-01 trial showed that OncoPSM accurately reconstructed IPD from KM curve, with RMSE below 0.004 for all curves. Log-rank p-values for the experimental and control groups (PFS: <0.001; OS: 0.010) closely matched the original article (PFS: <0.001; OS: 0.010). Hazard Ratios (HR) from reconstructed IPD data (PFS: 0.504 [0.4–0.63], OS: 0.731 [0.57–0.93]) were consistent with the original paper (PFS: 0.49 [0.39–0.61], OS: 0.73 [0.57–0.93]). The Log-logistic model provided the optimal fit for both PFS and OS curves according to the Akaike Information Criterion (AIC). Extrapolating the survival to a 10-year horizon, we created the PSM, derived the average state probability per cycle, and calculated state-weighted costs. The incremental cost for the experimental group was ¥42,068, with incremental quality-adjusted life years (QALYs) of 0.35, resulting in an ICER of 121,402, significantly below the willing-to-pay (WTP) threshold of 268,200 RMB/QALY. Uncertainty analysis showed a 99.7% probability of the experimental group being cost-effective.

**Conclusion:**

OncoPSM provides convenient treatment-cycle-based cost analysis, addressing the complexities of treatment costs in oncology research. By visualizing the entire CEA process, OncoPSM enables decision-makers to make informed decisions based on both statistical and intuitive assessments.

## Introduction

The rapid progress in oncology therapeutics, marked by the advent of personalized medicine and innovative targeted therapies, has inaugurated a new era in cancer treatment. However, these advancements bring significant financial challenges to healthcare systems globally. The imperative to balance clinical effectiveness with economic sustainability has never been more pronounced (Lichtenberg, 2020). Cost-effectiveness analysis (CEA) stands out as a pivotal tool, offering a structured approach to assess whether the health benefits of a new treatment justify its costs compared to existing standards of care (Ademi et al., 2013). Through CEA, decision-makers can optimize the allocation of limited healthcare resources, ensuring optimal patient care while managing healthcare expenditure growth.

CEA tools are indispensable for healthcare decision-makers, empowering them to make informed decisions regarding resource allocation (Seo and Cairns, 2021). Various tools and software packages are available to model, simulate, and analyze the costs and outcomes associated with different interventions. The CEA model framework can be configured in several ways, including Decision Tree (Gurusamy et al., 2017), State-Transition (Markov Cohort) Model (Peersman et al., 2014), and Partitioned Survival Model (PSM) (Tikhonova et al., 2017). Excel is one of the most widely accessible tool that can be used for basic CEA, it is often used in conjunction with other specialized software for data analysis and visualization (Buyukkaramikli et al., 2019). TreeAge Pro, which is a commercial software, is a versatile decision analysis software for creating decision trees, Markov models, and Monte Carlo simulations (Jiang et al., 2019). R packages such as rdecision (https://github.com/cran/rdecision), dampack (https://github.com/DARTH-git/dampack), BCEA (https://gianluca.statistica.it/books/bcea/), heemod (Filipović-Pierucci et al., 2017) and hesim (Zhang H. et al., 2023) are designed for health economic evaluation. They provide frameworks for conducting CEA and other types of economic evaluations using simulation-based approaches. By programming, researchers could achieve high level of customization and flexibility in data manipulation, model building, and statistical analysis.

However, most of these tools are designed for general clinical trials where treatment costs are often approximated using average values across the entire treatment period. While this approach is convenient, it fails to capture the significant variability in costs that can occur within individual treatment cycles, particularly in oncology, where treatment regimens are characterized by complex and dynamic dosing schedules (Zhang H. et al., 2023; Presa et al., 2023; Hui et al., 2023). To refine cost analysis in oncology cost-effectiveness modeling, it is essential to move beyond average costs and adopt a more granular consideration of treatment-cycle specific costs. By capturing the variability and true economic value of therapies, these refined approaches can lead to more accurate and meaningful cost-effectiveness evaluations, ultimately supporting better-informed clinical decision-making and healthcare policy.

We present oncoPSM, an interactive tool tailored for cost-effectiveness analysis in oncology trials using the Partitioned Survival Model (PSM). OncoPSM offers precise, efficient, and visually intuitive methods for computing research costs in oncology. We have also integrated the entire cost-effectiveness analysis pipeline, starting from reconstructing IPD, through fitting parametric survival models, to creating PSM models. Furthermore, we have visualized key aspects of the analysis process, enhancing researchers’ capacity to comprehend and intuitively evaluate the accuracy of results.

## Materials and methods

### The user-interface of oncoPSM

The oncoPSM presents an interactive online analysis tool developed, accessible at http://sw2-primary1.xiyoucloud.pro:13471/oncoPSM/. The user interface (UI) features four primary pages listed in the left sidebar: Parameters, Analysis, Document, and Contact. Users are encouraged to begin by reviewing the documentation, followed by inputting their custom parameters and submitting them for analysis. The parameter page consists of three main sections: parameters for IPD reconstruction, cost analysis, and PSM creation. Once all parameters are set, simply click the submit button at the bottom of the page to proceed with the analysis. The Analysis page primarily displays the key analysis results, the Incremental Cost-Effectiveness Ratio (ICER), alongside visualization of detailed processes including survival analysis, efficacy analysis, and cost analysis.

### Extracting data from published Kaplan-Meier survival curves

Data points can be extracted from Kaplan-Meier survival curves (KM Curves), where the x-axis represents time and the y-axis indicates survival probability. While we did not include this step in our analysis, various software options are available to digitize these graphs. Commonly used software includes DigitizeIt (http://www.digitizeit.de/), ScanIt (https://www.amsterchem.com/scanit.html), and WebPlotDigitizer (https://automeris.io/WebPlotDigitizer/). Although data extracted using different methods generally produce similar results (Liu et al., 2021), it is essential to manually verify the similarity between the original KM curve and the reconstructed KM curve to ensure the accuracy of estimation.

### Reconstructing individual patient data (IPD) from extracted survival data

For reconstructing IPD from the extracted survival data, we employed the R package IPDfromKM. This package utilizes an iterative algorithm adapted from the iKM method (Liu et al., 2021; Guyot et al., 2012). To assess the accuracy of the reconstruction, we provided statistical summary and KM curves reconstructed for visual evaluation. The statistical summary includes metrics such as root mean square error (RMSE), maximum absolute error, and mean absolute error per curve. Notably, the following thresholds are commonly utilized to signify the adequacy of the extracted data points for subsequent analyses: RMSE ≤0.05, mean absolute error ≤0.02, and maximum absolute error ≤0.05. Meanwhile, the reconstructed KM curve is presented for manual comparison with the original KM curve.

### Fitting parametric survival functions

To extrapolate the survival curve, the first step is to fit the Individual Patient Data with a full-parametric survival function, which involves selecting a suitable regression function for the time-to-event data. We incorporated six common accelerated failure time (AFT) models: Weibull, generalized Gamma, Log-Logistic, Log-Normal, Exponential and Gompertz. To assess the accuracy of the fitting, we utilized the Akaike Information Criterion (AIC), which evaluates the goodness of fit of the parametric function to the observed data while penalizing the number of parameters. The model with the smallest AIC was selected for both progression-free survival (PFS) and overall survival (OS), respectively.

### Creating partitioned survival model and conducting Monte-Carlo simulation

Utilizing the full-parametric survival function, we established a three-state PSM comprising stable disease (SD), progressive disease (PD), and death states. The PSM estimates the probability of a patient being in each health state at any given time under a specific therapy, by comparing the AUC (area under the curve) of KM curve between PFS and OS. Alongside the survival function, utility is a critical parameter in PSM creation, with values typically derived from literature focusing on similar contexts. We introduced a beta distribution to simulate utility values within a 20% range, consistent with prevailing literature practices (Nafees et al., 2017; Cheng et al., 2024; Zhang M. et al., 2023; Wang H. et al., 2023; Huo et al., 2023). For Monte-Carlo simulation, which involves multiple model iterations, users can define custom cycle lengths (weeks/cycle) and utility values for each survival state (SD, PD). The lifetime horizon is automatically calculated based on the user-input cycle length and the total number of treatment cycles in the cost data, ensuring flexibility for diverse trial designs. The lifetime horizon denotes the timeframe considered in the pharmacoeconomic model and significantly influences ICERs, which tend to decrease (indicating enhanced cost-effectiveness) with longer horizons. Model cycle represents the period used for calculating immediate state-transition probabilities, conventionally set at 3 weeks (Zhang M. et al., 2023; Wang H. et al., 2023; Huo et al., 2023). We then estimated three transition probabilities within each model cycle throughout the lifetime horizon: the probability of PD observed before death (p_sd.pd_), the probability of death from the SD (p_sd.d_), and the probability of death from the PD (p_pd.d_). By simulating this process N times, we can derive statistical distributions of costs and effects.

### Analyzing treatment costs across survival states and treatment cycles

Costs are categorized into State-Dependent Costs and State-Independent Costs. State-Dependent Costs capture medication expenses associated with disease states, while State-Independent Costs include all other care-related costs. Utilizing these user-defined costs per treatment cycle for each survival state, our approach initially involved simulating the cost distribution N times using a gamma distribution. This process generated a four-dimensional matrix comprising treatment group, survival state, treatment cycle, and simulation time. Leveraging the results obtained from the PSM, we extracted state probabilities for each model cycle. Crucially, we aligned the model cycle with the treatment cycle, enabling the calculation of survival state-weighted costs per model cycle. To address potential bias in cost attribution due to events occurring within model cycles, we applied half-cycle correction. Finally, we computed the present value of costs by discounting the model cycle costs with the specified discount rate.

### Decision analysis and uncertainty quantification

With the previously computed fitted parametric survival function, PSM, and user-inputted survival-state specific utilities, we can calculate the effectiveness metric, QALYs, and its distribution across N simulation runs. Utilizing the precomputed survival-state-weighted costs, we derive the core metric for cost-effectiveness analysis, the ICER. This ratio represents the difference in incremental cost divided by the difference in incremental effect, with a statistical distribution generated from Monte-Carlo simulation. To facilitate decision-making, we evaluated the magnitude of the ICER, typically against a willing-to-pay (WTP) threshold. According to The Guidelines of Pharmacoeconomic Evaluations of China (2020) (Liu et al., 2020), the threshold is set as 3×GDP for ‘cost-effective’ interventions, with 1×GDP used as a strict benchmark for ‘highly cost-effective’ classifications. Additionally, other metrics such as net monetary benefit (NMB) and incremental NMB (iNMB) are inferred and reported with the assistance of WTP. Probability Sensitivity Analysis (PSA) is the standard methodology for quantifying the impact of parameter uncertainty. In our analysis, we have integrated beta and gamma distributions for the two critical parameters, utility and cost, respectively. Notably, as our cost varies with the treatment cycle and is not fixed, it is not suitable for univariate sensitivity analysis.

### Data visualization

We thoroughly visualized the entire analysis process, which sequentially includes the statistical evaluation of IPD reconstruction, the KM curve of the reconstructed IPD, accuracy assessment in fitting the parametric survival function, the parametric survival function after Monte-Carlo simulation, changes in survival state probability over treatment cycles, changes in survival state-weighted costs over treatment cycles, and the assessment of cost-effectiveness. This included the cost-effectiveness plane, cost-effectiveness acceptability curve (CEAC), and the expected value of information (EVPI) plot. All visualizations were implemented using the R packages ggplot2 and survminer.

## Results

### Data extraction and processing from KM curves of CHOICE-01 trial

An example using oncoPSM was provided based on the CHOICE-01 trial, a multicenter randomized phase III study. This clinical trial compared Toripalimab plus chemotherapy (experimental group) versus chemotherapy alone (control group) for treatment-naïve advanced non–small-cell lung cancer (NSCLC) (Wang J. et al., 2023) The experimental group enrolled 309 patients, while the control group enrolled 156 patients. The study reported PFS data up to 30 months and OS data up to 42 months. The total PFS events were 194 in the experimental group and 132 in the control group. The total OS events were 17 in the experimental group and 108 in the control group. We extracted datapoints of PFS and OS for both the experimental and control groups using WebPlotDigitizer (Supplementary Tables S1-S4). To reconstruct the IPD more accurately, we also used risk tables of each survival curve as input (Supplementary Table S5).

### Cost composition in CHOICE-01 trial

The cost composition in this study is quite complex due to the treatment regimens that adjust over time and with disease state. Briefly, patients received first-line therapy every 3 weeks for 4-6 cycles, followed by maintenance therapy until disease progression, death, unacceptable toxicity, investigator decision, withdrawal, or completion of 2 years of treatment, whichever occurred first. Based on this, we collected and organized direct drug costs according to treatment cycle and disease state (Supplementary Tables S6-S7). Since the trial did not specify treatment details after disease progression, we set the cost after PD to 0 for all groups for convenience. Indirect costs resulting from tumor assessments are also complex. The study reported that computed tomography/magnetic resonance imaging were performed at baseline, every 6 weeks during the first 12 months, and every 9 weeks thereafter. Given that indirect costs generally do not vary with survival state, we consolidated all indirect costs—including imaging, lab exams, and supportive care—into a single table, with each row representing a treatment cycle and each column representing a type of indirect cost (Supplementary Table S8). To facilitate researcher, a template pre-loaded with the data used in this example can be downloaded from https://github.com/dev01ontheway/oncoPSM/tree/main/oncoPSM_demoData.

Another important parameter to consider is the time unit. Many studies, including CHOICE-01, use months as the unit for KM curves. However, CEA often uses QALYs to measure effect, and utility values are typically calculated on an annual basis. Therefore, it is necessary to convert the time units accordingly. Additionally, the times of simulations significantly impacts memory usage.

### IPD reconstruction and parametric survival function fitting

**FIGURE 1:**
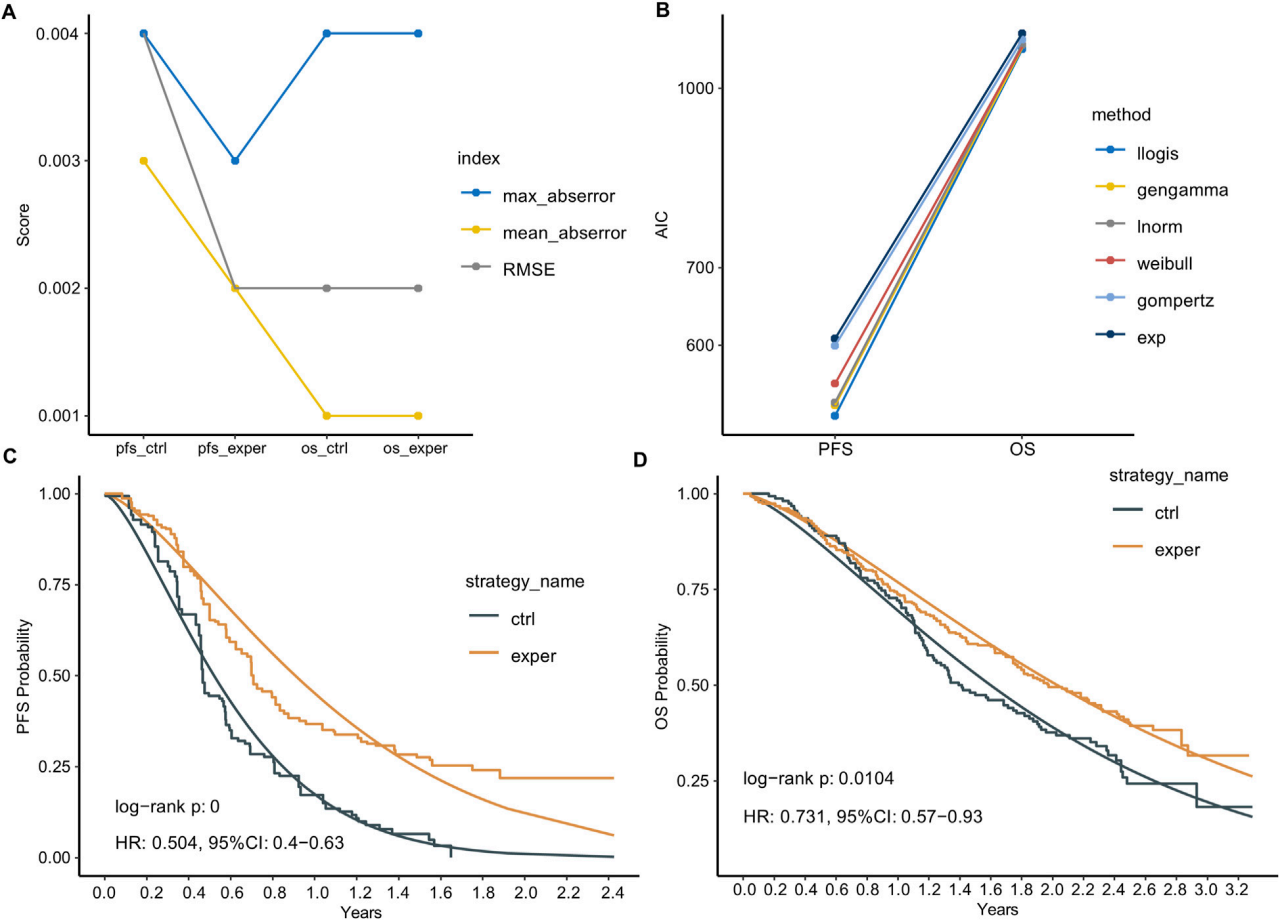
IPD reconstruction and parametric survival function fitting. **(A)** Statistical summary of the reconstructed IPD compared to the extracted data. **(B)** Akaike Information Criterion (AIC) of six common parametric survival models used to fit the survival data. Llogis: Log-logistic; gengamma: generalized gamma; lnorm: log-normal; Weibull: Weibull distribution; gompertz: Gompertz; exp: Exponential. **(C,D)** Reconstructed Kaplan-Meier (KM) curves and the Log-logistic fitted curves for PFS **(C)** and OS **(D)**.

Based on the data points extracted from the KM curves, we reconstructed the IPD. We first assessed the accuracy of our reconstruction using three metrics: root mean square error (RMSE), maximum absolute error, and mean absolute error. All three metrics for the four curves were less than 0.004, which is significantly better than the recommended standards, indicating high reconstruction accuracy (Figure 1A). Notably, the PFS curve in the control group had slightly poorer metric values, possibly due to the number of data points we extracted.

Next, we plotted KM curves for PFS and OS using the reconstructed IPD data (Figures 1C,D). Comparing the reconstructed KM curves to those published in the original article, we observed a high degree of similarity, indicating no significant reconstruction bias. We also calculated the log-rank p-values for the experimental and control groups using the reconstructed IPD data. For PFS, the p-value was <0.0001, and for OS, it was 0.0104. These results are very similar to the original article’s p-values of <0.0001 for PFS and 0.0108 for OS. Additionally, the Hazard Ratios (HR) calculated using the reconstructed IPD data were 0.504 [0.4–0.63] for PFS and 0.731 [0.57–0.93] for OS. These are closely aligned with the original paper’s reported values of HR: 0.49 [0.39–0.61] for PFS and 0.73 [0.57–0.93] for OS.

We employed six common parametric survival models to fit the survival data, with the Akaike Information Criterion (AIC) serving as the goodness-of-fit indicator. The Log-logistic model demonstrated the best fitting performance for both PFS and OS curves (Figure 1B). We compared the reconstructed KM curves with the Log-logistic fitted curves for PFS and OS (Figures 1C,D). Interestingly, the parametric fitting exhibited better performance for the OS curve compared to the PFS curve, likely attributed to the longer follow-up period for OS. Numerically, the median PFS times calculated from the parametric function were 0.52 and 0.90 years in the control and experimental groups, respectively, slightly longer than those reported in the original paper (0.45 and 0.70 years). Similarly, the median OS times calculated from the parametric function were 1.60 and 2.03 years in the control and experimental groups, respectively, closer to the reported values (1.42 and 1.98 years).

### Estimating survival state probability per treatment cycle

**FIGURE 2:**
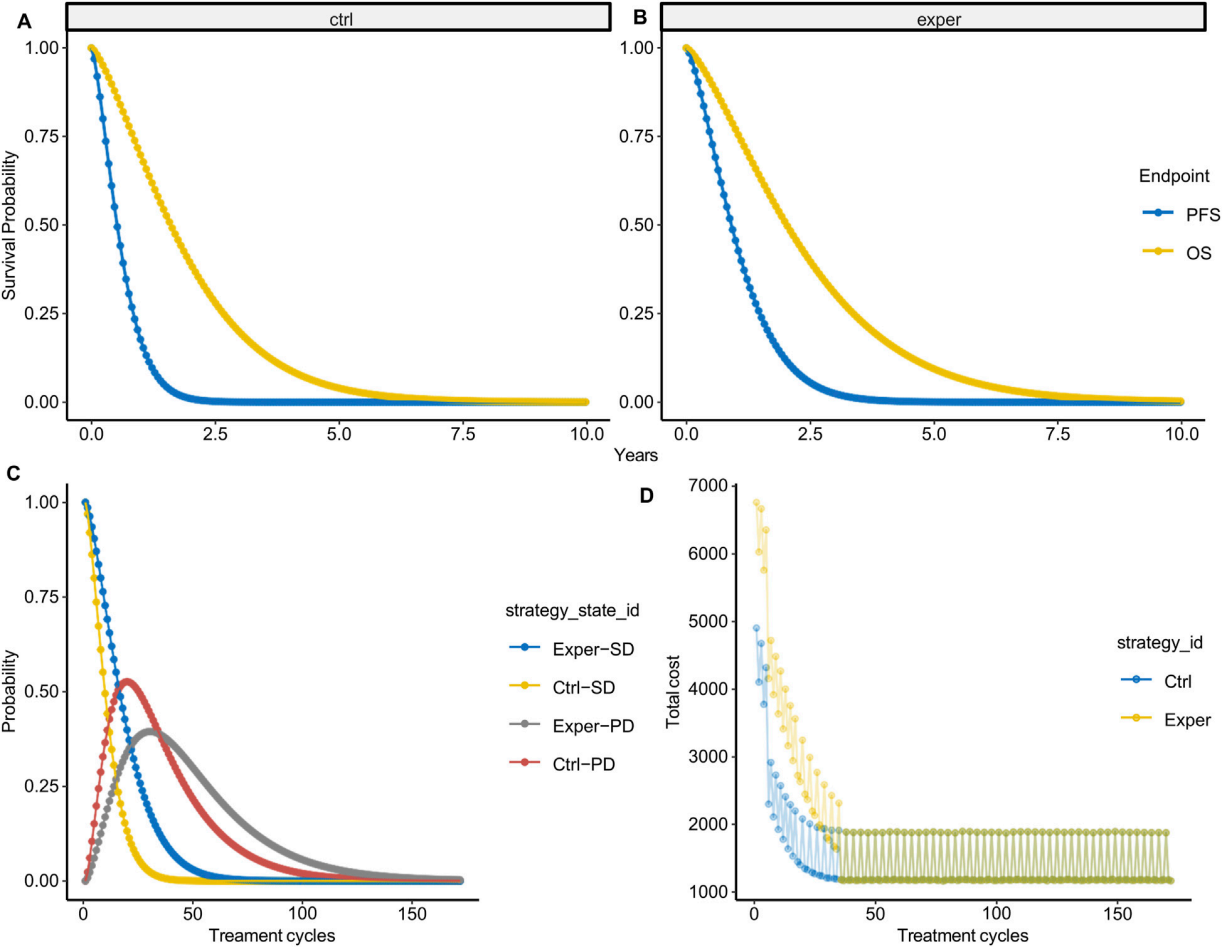
State probability per treatment cycle. **(A,B)** Average probabilities of PFS **(A)** and OS **(B)** over 10 years based on 1,000 simulations. **(C)** State probability per treatment cycle in the experimental and control groups. **(D)** State-weighted cost per treatment cycle in the experimental and control groups.

With the parametric survival function fitted, we extrapolated the survival curve to a lifetime horizon derived from dynamic calculation (e.g., 10 years in this case, based on 172 cycles × 3 weeks/cycle). According to the survival function, at 10 years, 0.02% and 0.22% of patients are still alive in the control and experimental groups, respectively. Following multiple simulations, we derived the average survival probability during lifetime horizon (Figures 2A,B). In the control group, nearly all patients progressed at 1.5 years and succumbed to the disease by 5 years, while in the experimental group, progression occurred at 2.5 years with a subsequent survival until 7.5 years. We then inferred survival state probability in each model cycle thereafter (Figure 2C). The probability of SD state decreased with each treatment cycle, with the experimental group displaying notably higher SD state probability during the first 50 treatment cycles compared to the control group. Conversely, the probability of PD state increased during the first ∼25 treatment cycles before decreasing. The experimental group exhibited lower PD state probability during the first 36 treatment cycles but higher PD state probability thereafter.

With survival state probability per model cycle available, we calculated the survival state-weighted cost per treatment cycle (Figure 2D). It is noteworthy that the cost fluctuates with the treatment cycle, partly due to the introduction of cost probability distributions. However, more importantly, this variability reflects the influence of imaging costs within indirect costs, further emphasizing the ability of our cost model to accurately reflect cost fluctuations.

### Decision analysis and uncertainty assessing

The primary metric was the ICER, which stands at 121,402 RMB/QALY in our dataset. Correspondingly, the incremental cost is 42,067.86 (35,624.7–48658.39), and the incremental QALY is 0.3465 (95%CI: 0.2100–0.4783). Since the ICER was substantially lower than the WTP threshold of 268,200 RMB/QALY, the conclusion was that the experimental intervention was cost-effective.

**FIGURE 3:**
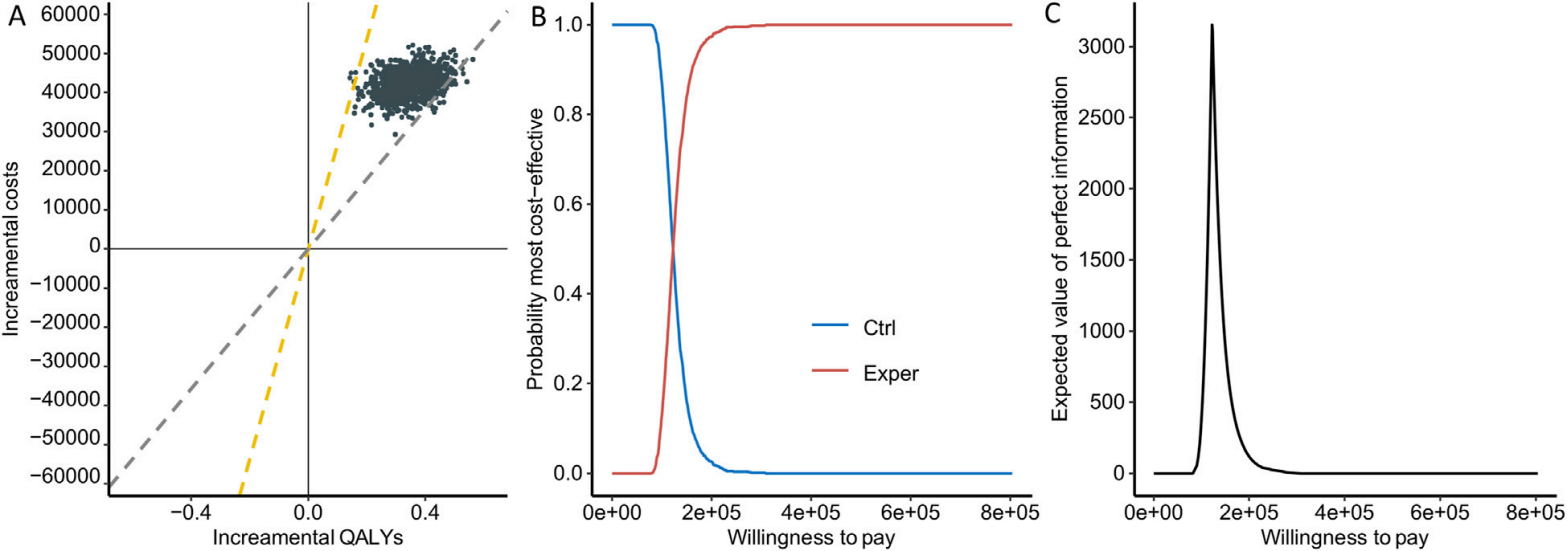
Uncertainty assessment. **(A)** Cost-effectiveness planes. Each point represents the result of one simulation, with the yellow line indicating 3 times GDP and the gray line indicating 1 time GDP. **(B)** Cost-effectiveness acceptability curves. The red curve indicates higher cost-effectiveness for the experimental group, while the blue curve indicates higher cost-effectiveness for the control group. **(C)** Expected value of information plot.

To assess the uncertainty introduced by the parametric distribution, we utilized cost-effectiveness planes, cost-effectiveness acceptability curves (CEACs), and the expected value of information (EVPI). Among the 1,000 simulation runs, the experimental group lacks cost-effectiveness in only 3 instances (0.3%), i.e., experimental group showed a 99.7% probability of being a cost-effective option at a WTP threshold of 268,200 RMB/QALY (Figure 3A). Comparatively, when using 1-fold GDP as WTP cutoff, the experimental group exhibited a 6.1% probability of being cost-effective. From the CEACs, which depict the probability that each treatment strategy is the most cost-effective, we observed a crossover between the two curves at a WTP threshold of approximately 122,000 RMB, aligning closely with the value of the ICER. This represents the point where both the experimental and control groups demonstrated similar levels of cost-effectiveness (Figure 3B). The Expected Value of Perfect Information (EVPI), which integrates the probability of being most effective with the magnitude of expected Net Monetary Benefit (NMB), reaches its peak at 3,208.91 RMB when the WTP equals 122,000 RMB (Figure 3C).

## Discussion

We developed OncoPSM, an interactive tool for cost-effectiveness analysis using partitioned survival models in oncology trials. The main value of the tool lies in its ability to address the complexities of treatment-cycle-specific cost analysis in oncology trials. Previously, most software utilized average costs for estimation when using the PSM model. However, this approach is overly simplistic for oncology research, where treatment regimens are often multifaceted, making cost calculations challenging. Direct drug costs include first-line, maintenance, and second-line treatments, while indirect costs such as imaging and laboratory expenses exhibit periodicity. Therefore, in the development of OncoPSM, we considered these factors. We integrated survival state and treatment-cycle-specific costs as inputs, incorporating probability distributions to comprehensively assess cost uncertainty. By using the model-cycle-specific state probabilities obtained from PSM as weights and discounting with a discount rate, we derived the final costs. In theory, our algorithm should yield costs much higher than average costs, as initial treatment phases typically incur higher direct drug costs, which are reflected in the discounted costs. In addition to addressing the challenges of cost calculation, we also integrated the entire cost-effectiveness analysis process, including IPD reconstruction, parametric survival function fitting, PSM construction, and ICER calculation. Furthermore, our tool incorporates a series of quality control metrics at key stages, including RSEM for IPD reconstruction effectiveness, AIC for parametric survival function fitting effectiveness, and probability sensitivity analysis, and uncertainty visualization. Moreover, we visualized the entire process, enabling researchers to intuitively evaluate the analysis results.

In the CHOICE-01 study, we validated the performance of our tool. We arrived at a conclusion consistent with other studies (Zhang M. et al., 2023; Wang H. et al., 2023; Huo et al., 2023), indicating the cost-effectiveness of toripalimab combined therapy. However, we differed significantly from previous research in three key aspects. Firstly, our effect analysis utilized the latest and mature OS data, whereas previous studies relied on mid-term and immature OS data, leading to a higher calculated incremental effect (Zhang M. et al., 2023; Wang H. et al., 2023; Huo et al., 2023). Secondly, we conducted statistical indicator calculations and visualizations at two critical analysis stages, IPD reconstruction and parametric survival function fitting, enabling researchers to assess study accuracy logically and intuitively. Thirdly, and the most importantly, in our cost analysis, the unit price of toripalimab was 1,884.86 RMB/240 mg, obviously lower than in many previous studies (2003 RMB/240 mg in Wang H. et al. (2023); 2,717 RMB/240 mg in Huo et al. (2023); 2,780 RMB/240 mg in Zhang M. et al. (2023), yet our calculated incremental cost was only slightly lower than in previous studies. This discrepancy is evidently due to differences in cost analysis methodologies. We provided clarity at every step of the cost analysis and visualized state-weighted costs, enabling researchers to intuitively judge the accuracy of the cost analysis, which was somewhat lacking in previous studies. Furthermore, we tested calculating ICER using the average cost of all cycle (2,162 RMB in the experimental group, 1,778 RMB in the control group), yielding an ICER of 3,308 RMB (with corresponding incremental cost of 1,138 RMB). This result aligned with our expectations, as average cycle costs significantly underestimated actual costs. We further tested calculating ICER using the cost of first-line treatment (4,907 RMB in the experimental group, 3,023 RMB in the control group), assuming patients would continue with the experimental treatment until progression, resulting in an ICER of 11,282 RMB (with corresponding incremental cost of 3,905 RMB). This finding was somewhat unexpected, as the ICER calculated using the cost of first-line treatment was lower than that obtained by calculating costs per cycle. Upon investigation, we discovered that maintenance therapy accounted for the most significant and longest-lasting cost difference between the experimental and control groups (maintenance therapy costs were 3,411 and 1,527 RMB in the experimental and control groups, respectively). This underscores the complexity of cost analysis, where neither the cost of first-line treatment nor maintenance therapy costs alone suffice to reflect true treatment costs; calculating costs per cycle emerges as the optimal choice.

Given that this tool is primarily developed for oncology research, it may not necessarily be suitable for other types of studies. Firstly, we utilize PSM, which is only applicable to clinical trials with survival as the endpoint. Other clinical trials with disease outcomes as the endpoint may use state transition models such as cDTSTM, iDTSTM, etc. Secondly, to facilitate researchers’ quick adoption, we have set some default options commonly used in oncology clinical trials in the tool, including treatment cycles every 3 weeks (Q3W). Meanwhile, users can also customize cycle lengths, and the lifetime horizon is dynamically calculated based on the inputted cycle length and the number of treatment cycles, enhancing flexibility for diverse research scenarios. Thirdly, and most importantly, our cost analysis requires researchers to customize the cost for each treatment cycle, including both direct drug costs and indirect costs such as imaging, examinations, and supportive care. Therefore, if costs do not vary with treatment cycles, there may be no need to use this model, and conventional R software like heemod and hesim may be more propriate.

The tool, originating from the need to address cost analysis in oncology clinical trials, integrates the entire process of cost-effectiveness analysis, considering numerous factors that influence QALYs and costs. However, there are still some areas that require improvement. Firstly, we have adopted a default treatment cycle of Q3W. If there is a significant demand from users to set custom treatment cycles in practice, we will consider opening up customization options. Secondly, the PSM consumes considerable memory. With a simulation count of 1,000, the average memory consumption approaches 150 GB. This may cause lagging when a large number of users are using the tool simultaneously. We will refine memory consuming to improve this in the future. Considering that there may be some aspects we have not fully considered, we have also provided access to all data during the analysis process, including key parameters of the parametric survival function, iNMB, and other parts not extensively explained in our article.

We have developed and validated OncoPSM, an interactive tool specifically tailored for conducting cost-effectiveness analyses in oncology clinical trials. OncoPSM addresses the intricacies of cost analysis arising from complex treatment regimens, thereby enhancing the accuracy and user-friendliness of CEA. By integrating a comprehensive analysis pipeline and enabling real-time analysis, OncoPSM aims to empower users to make more informed decisions regarding the adoption of new cancer therapies. Future research is needed to validate the reliability and generalizability of OncoPSM by comparing its performance with established CEA methods and widely used tools.

## Data Availability

The original contributions presented in the study are included in the article/Supplementary Material, further inquiries can be directed to the corresponding author.
